# The Application of Complexity Analysis in Brain Blood-Oxygen Signal

**DOI:** 10.3390/brainsci11111415

**Published:** 2021-10-27

**Authors:** Xiaoyang Xin, Shuyang Long, Mengdan Sun, Xiaoqing Gao

**Affiliations:** Center for Psychological Sciences, Zhejiang University, Hangzhou 310012, China; xiaoyangxin@zju.edu.cn (X.X.); 21939009@zju.edu.cn (S.L.); mengdansun@zju.edu.cn (M.S.)

**Keywords:** complexity analysis, blood-oxygen signal, biomarker, entropy

## Abstract

One of the daunting features of the brain is its physiology complexity, which arises from the interaction of numerous neuronal circuits that operate over a wide range of temporal and spatial scales, enabling the brain to adapt to the constantly changing environment and to perform various cognitive functions. As a reflection of the complexity of brain physiology, the complexity of brain blood-oxygen signal has been frequently studied in recent years. This paper reviews previous literature regarding the following three aspects: (1) whether the complexity of the brain blood-oxygen signal can serve as a reliable biomarker for distinguishing different patient populations; (2) which is the best algorithm for complexity measure? And (3) how to select the optimal parameters for complexity measures. We then discuss future directions for blood-oxygen signal complexity analysis, including improving complexity measurement based on the characteristics of both spatial patterns of brain blood-oxygen signal and latency of complexity itself. In conclusion, the current review helps to better understand complexity analysis in brain blood-oxygen signal analysis and provide useful information for future studies.

## 1. Introduction

One of the distinct features of the brain is its daunting complexity, which remains a popular research topic [1]. Dating back to the 1930s, Sigmund Freud proposed to investigate the relation between mental illness and brain complexity but eventually failed due to the lack of reliable analysis methods [2]. After continuous exploration and development, an academic discipline called complexity science emerged. As the concepts and methodology from mathematics, physics, and computer sciences were introduced into the field of psychology, complexity science also had its contribution in quantifying the behavioral and emotional problems of patients with mental disorders in the past two decades. For example, Paulus et al. [3] found that compared with healthy controls, schizophrenic patients showed more predictable behavior (i.e., less complex) during a consecutive binary choice task. Similarly, researchers found that self-rated consecutive daily record of mood completed by patients with bipolar disorder exhibited a more organized pattern (i.e., less complex) than healthy controls [4]. In many cases, aging and diseases often come along with loss of physiological complexity [5,6]. With the development of brain imaging techniques in recent years, brain blood-oxygen signal has been widely used as an objective indicator of brain activity. Thus, brain signal complexity has been used as an external and objective indicator of complexity of brain activity. In this paper, we provide a review of recent studies (since 2010) measuring complexity of blood-oxygen signal and discuss opportunities and challenges in this field.

## 2. Complexity of Brain Blood-Oxygen Signal

### 2.1. Physiological Complexity of Brain

**Figure 1:**
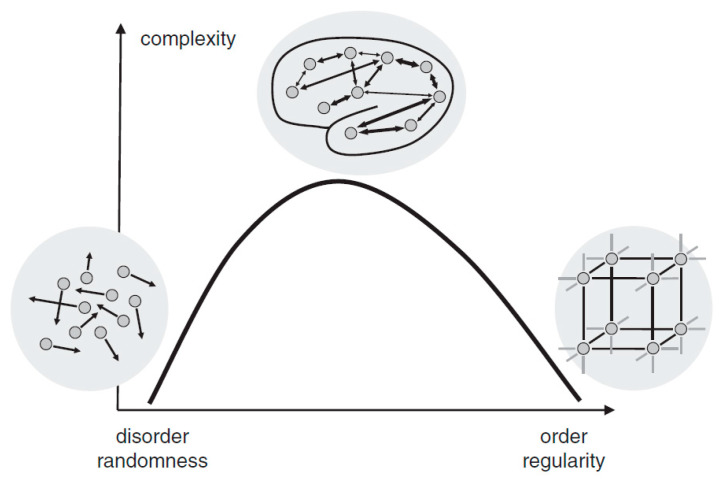
The relationship between randomness and complexity. Reprinted with permission from ref. [10]. Copyright 2015 John Wiley and Sons.

Complexity is a characteristic of a system and is inherently inseparable from it. Intuitively, a complex system can be described as a system that consists of many interacting parts, for example, the metro systems of New York City. The human brain, which is composed of innumerable neurons and synapses, undoubtedly exhibits its astonishing complexity. Although there is an ongoing debate on how to define and measure complexity mathematically, two basic views proposed by Herbert Simon [7] have been widely adopted. First, most of the complex systems are hierarchically organized and can be decomposed into subsystems and interactions among them. Second, complexity is not randomness. In fact, it is a mixture of randomness and regularity. As illustrated in Figure 1, either highly ordered (e.g., crystal) or highly random (e.g., gas) systems are of low complexity. A system, however, when showing coexistence of both order and randomness (e.g., the human brain), exhibits high complexity. As Simon suggested [7], the physiological complexity of the brain does not only arise from the interaction of numerous neuronal circuits that operate over a wide range of temporal and spatial scales, but is also inextricably bound to its unique physiological structure for the coexistence of randomness and regularity [8,9].

### 2.2. Measuring Brain Complexity through Brain Blood-Oxygen Signals

Despite the definition of complexity by Simon, quantifying brain complexity directly is still difficult. This is because deducting the whole brain into a myriad of subsystems is extremely resource and time consuming, and it appears to be unpractical in application. Therefore, researchers use the complexity of brain neurophysiological outputs as an indirect measure of brain complexity.

Blood-oxygen signal is a common brain neurophysiological output, which reflects changes in cerebral blood flow and oxygen consumption resulted from brain activities. One of the most widely used signals is the blood oxygen level-dependent (BOLD) signal obtained by functional magnetic resonance imaging (fMRI). Besides, there are other kinds of blood-oxygen signals such as oxyhemoglobin (O2Hb) and deoxyhemoglobin (HHb) signals obtained by functional near-infrared spectroscopy (fNIRS). The complexity of brain blood-oxygen signal can be used as a valid estimate of physiological complexity of the brain. The majority of brain blood-oxygen complexity measurements are entropy-based algorithms [11] such as approximate entropy (ApEn), sample entropy (SampEn) and permutation entropy (PE). For different types of complexity measurements, the algorithms vary. However, in general, they can be evaluated with Equation (1):

(1) $$Complexity_{category}\left(pos\right)=Cal\_Complexiy_{catetory}\left[position,parmeters,siganl\right]$$

Category defines the type of algorithm. Parameters specify the required parameters for a specific complexity algorithm. Position denotes the location information: for BOLD signal obtained with fMRI, position represents a certain voxel location; for O2Hb and HHb signals, position represents the corresponding location of a certain channel. When the necessary information (category, position, parameters, and signal) is given, the complexity value can then be calculated. Specifically, if the complexity algorithm is a certain type of entropy, the whole brain complexity pattern, or in other words, brain entropy map (BEM) can be obtained by calculating entropy value at each spatial location.

## 3. Current Studies in Complexity of Brain Blood-Oxygen Signals

Table 1 summarizes studies on complexity of blood oxygen signals since 2010. These studies can be divided into two categories: one is biomarker-oriented studies aiming to apply complexity measures of brain blood-oxygen signals as a biomarker to characterize patient populations. The other is methodology-oriented studies aiming to develop measurements of brain blood-oxygen signal complexity. Based on aims of the studies from these two categories, this chapter aims to review current literatures in the following three aspects.

### 3.1. Brain Blood-Oxygen Signal Complexity as a Biomarker

Complexity is a nature of a system and inherently inseparable from it. According to the loss of physiological complexity theory, brain physiological complexity is reduced in many disease states and aging, reflected in the reduction of blood-oxygen signal complexity [6,12,13,14]. Findings from many studies on resting-state brain blood-oxygen activities are consistent with this theory as shown in Table 1. For example, Sokunbi et al. [15] found that ADHD patients showed lower mean whole brain SampEn than healthy controls in resting state. In another similar study, Sokunbi [16] found the mean whole brain SampEn of young adults is significantly higher than older adults in resting state. In most cases, however, significant whole brain BOLD SampEn differences were not observed. Therefore, researchers usually take advantage of the high spatial resolution BOLD signals obtained with fMRI to search for the specific regions that exhibit significant differences. Yang et al. [17] found that MSE of BOLD signals in posterior cingulate gyrus and hippocampal cortex in older adults were significantly lower than that of younger adults. Liu et al. [18] found Alzheimer patients showed a significant reduction in ApEn of BOLD signals in anterior cingulate cortex and left precuneus.

However, some task-state studies indicated that aging or illness does not necessarily reduce the brain blood oxygen complexity. For example, Sokunbi et al. [19] found when performing Cyberball social exclusion task, the whole brain SampEn in schizophrenic patients was significantly higher than that of normal participants. Gu et al. [20] found that compared with normal controls, the permutation entropies (PEs) of BOLD signal in dorsolateral prefrontal cortex were significantly higher in patients with ADHD during a working memory task.

**Figure 2:**
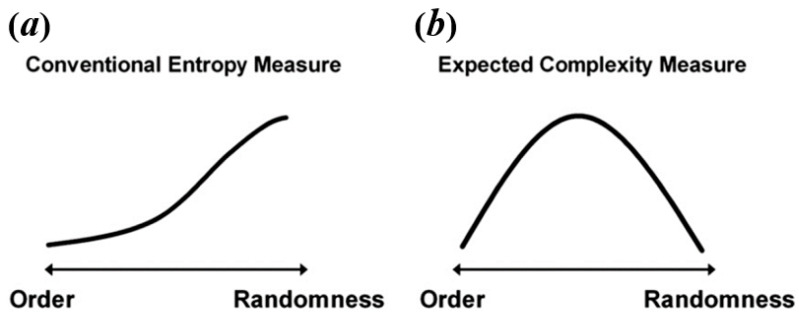
The relation between level of randomness and conventional entropy measure (**a**) and expected complexity (**b**). Reprinted with permission from ref. [49]. Copyright 2021 Elsevier.

Some researchers consider that resting-state BOLD signal and task-state BOLD signal have different natures, so it explains that they may exhibit different temporal brain entropy (tBEN) patterns [20,21]. Here, however, we argue that the discrepancy in complexity patterns between resting-state and task-state may arise from the fact that many studies falsely equate complexity to entropy. As mentioned above, the value calculated by entropy algorithms can only be treated as an estimate of complexity, regardless of the type of algorithm. This is because when signals are of high randomness, the complexity would decrease but the brain entropy measured by common entropy algorithms shows the opposite trend as shown in Figure 2a. For example, in the studies of Sokunbi et al. [19] and Gu et al. [20], patients with mental illness showed lower task-elicited brain activation, leading to a higher randomness of signals, which in turn led to a higher entropy value.

**Table 1:** A summary of literatures on brain blood-oxygen complexity analysis in since 2010.

Measure	Signal Type	Res. Orientation	Participants	Main Findings	Ref
ApEn	Task-BOLD	Biomarker	Older adults (40)	Cognitive ability was positively correlated with regional brain BOLD complexity.	[22]
SampEn	Task-BOLD	Biomarker	ADHD (17); HC (13)	The mean whole brain BOLD complexity of ADHD group was significantly lower than the HC; the mean regional brain complexity values have a significant negative correlation with ADHD score.	[15]
MSE	Rest-BOLD	Biomarker	Older adults (99); Younger adults (56)	The mean whole brain BOLD complexity of younger adults was significantly higher than that of older adults; the high cognitive ability group showed significantly higher whole brain BOLD complexity than the low cognitive ability group; regional brain BOLD complexity was significantly correlated with cognitive function.	[17]
ApEn	Rest-BOLD	Biomarker	Younger adults (8); Older adults (8); fAD (22)	Brain BOLD complexity decreased with normal aging and cognitive decline.	[18]
SampEn	Task-BOLD	Biomarker	SZ (13); HC (16)	Brain BOLD complexity of SZ patients was higher than that of HC when performing Cyberball social exclusion task.	[19]
MSE	Rest-BOLD	Biomarker/ Methodology	Older adults (8); Younger adults (8)	Brain BOLD complexity was used to discriminate younger from older participants as well as grey matter from white matter.	[23]
SampEn	Rest-BOLD	Biomarker/ Methodology	Older adults (53); Younger adults (53)	SampEn was used to discriminate the younger from the elderly adults with short length data; the suggested value of *m* was 2.	[16]
SampEn	Rest-BOLD	Methodology	1049	Using a data-driven clustering method, the entire brain was organized into seven regional brain entropy networks that are consistent with known brain parcellation.	[24]
MSE	Rest-BOLD	Biomarker/ Methodology	20	Complexity of the BOLD signal showed different patterns from white, pink, and red noises; neural complexity across all networks was negative.	[25]
MSE	Rest-BOLD	Biomarker	SZ (105); HC (210)	Complexity of BOLD signals in SZ patients showed two patterns (toward either regularity or randomness), which were respectively associated with positive or negative symptoms of schizophrenia.	[26]
fApEn; SampEN	Rest-BOLD	Biomarker/ Methodology	86	Compared to SampEn, fApEn was better at discriminating different age groups and have shown to be a more sensitive method.	[27]
SampEn	Rest-BOLD	Biomarker	CPI (29); HC (29)	The BEN map of CPI patients demonstrated significant differences from HC, and altered functional connectivity patterns were associated with abnormal BEN regions.	[28]
SampEn	Rest-BOLD	Biomarker	RRMS (34); HC (34)	BOLD complexity of RRMS patients was significantly increased in some regions and was positively correlated with disease severity.	[29]
SampEn	Rest-BOLD	Biomarker	seafarers (20); HC (20)	BOLD complexity pattern of seafarers was significant different from HC.	[30]
PE	Rest-BOLD	Biomarker	MCI (65); AD (29); HC (30)	The BOLD complexity of AD patients was significantly lower than that of MCI patients and HC; that of AD patients and MCI patients was significantly correlated with ReHo in several brain regions associated with AD.	[31]
PE	Task-O2Hb	Biomarker	ADHD (15); HC (16)	BOLD complexity in the right dorsolateral prefrontal cortex of ADHD patients were significantly higher than that of HC.	[20]
SampEn; MSE	Rest-BOLD	Methodology	354	Proposed a generic strategy to minimize the relative error of SampEn to determine the appropriate complexity measurement parameters.	[32]
SampEn	Task-BOLD	Biomarker	CFS (43); HC (26)	Regional brain complexity in CFS patients was lower than that in HC when performing a Stroop task.	[33]
SampEn	Rest-BOLD Task-BOLD	Biomarker	CFS (45); HC (27)	BOLD complexity of CFS patients was higher in the default mode network at resting-state or performing a Stroop task.	[21]
SampEn	Rest-BOLD	Biomarker	892	BOLD complexity was positively associated with intelligence.	[34]
SampEn; MSE	Rest-BOLD	Biomarker	MCI (65); AD (29); HC (30)	BOLD complexity of AD and MCI were lower than HC; AD patients showed lower BOLD complexity than MCI.	[35]
MSE	Rest-O2Hb	Biomarker	MCI (65); AD (29); HC (30)	O2Hb complexity in AD patients was lower than HC and positive correlated with cognitive ability.	[36]
SampEn MSE	Task-O2Hb Task-HHb	Biomarker	AD (11); HC (11)	When performing memory-related tasks, O2Hb complexity of AD was higher than that of HC.	[37]
SampEn	Rest-BOLD	Biomarker	107	SampEn-CBF and SampEn-fALFF correlations were only observed in a few brain regions, demonstrating that complexity, CBF, and fALFF are independent brain activity measures.	[38]
SampEn	Rest-BOLD	Biomarker	ASD (20); HC (17)	BOLD complexity was negatively correlated with severity of ASD behaviors.	[39]
SampEn	Rest-BOLD	Biomarker	SZ (53); HC (59)	Compared with HC, SZ showed decreased brain BOLD complexity.	[40]
SampEn; MSE	Task-O2Hb	Biomarker	AD (11); HC (11)	AD showed significant differences from HC in O2Hb complexity during VFT and WM tasks.	[41]
SampEn	Rest-BOLD	Biomarker	Stroke patients (23); HC (19)	Stroke patients showed reduced BOLD complexity in the motor area.	[42]
MSE	Rest-BOLD	Biomarker	MCI (169); HC (176)	BOLD complexity in MCI was significantly lower than that in HC and correlated with severity of MCI.	[43]
MSE	Rest-BOLD	Biomarker	BP (125); SZ (107); SAD (98); HC (156)	Significant differences as well as overlaps of brain BOLD signal complexity between different psychotic disorder groups were found.	[12]
MSE	Task-O2Hb Task-HHb	Biomarker	15	Brain complexity during performing intentional memory task was significantly higher than that during purposefully forgetting.	[44]
MSE	Rest-O2Hb Rest-HHb	Biomarker	ASD (25); HC (22)	Brain complexity could be used to distinguish ASD from HC. Compared with HC, altered brain complexity in ASD is seen more in IFG than in TC and in left hemisphere than in right hemisphere.	[45]
MSE	Rest-BOLD	Biomarker	LLD (35); HC (22)	LLD patients showed decreased complexity only in the right posterior cingulate gyrus but increased complexity in affective processing, sensory, motor, and temporal nodes. Complexity in the left frontoparietal network partially mediated the relation between depression severity and the mental components of quality of life.	[46]

Note: ADHD: Attention deficit hyperactivity disorder; HC: healthy control; fAD: familial Alzheimer’s Disease; CPI: chronic primary insomnia; AD: Alzheimer’s Disease; CFS: chronic fatigue syndrome; ASD: Autism Spectrum Disorder; BP: psychotic bipolar disorder; SZ: schizophrenia; RRMS; relapsing-remitting multiple sclerosis; LLD: depression in later life; Task-BOLD: task blood oxygen level dependent signal; Rest-BOLD: rest blood oxygen level dependent signal; Task-O2Hb: task oxyhemoglobin signal; Rest-O2Hb: rest oxyhemoglobin signal; Task-HHb: deoxyhemoglobin; fALFF: fractional amplitude of low frequency fluctuation (refering to the ratio of power spectrum of low-frequency (0.01–0.08 Hz) to that of the entire frequency range [47]); ReHo: regional homogeneity (refering to Kendall’s coefficient concordance of BOLD signals in neighboring voxels [48]); BEN: brain entropy; tBEN: temporal brain entropy.

Thus, the increase of complexity observed by the above-mentioned task-state studies may be as a result of a higher randomness rather than true complexity.

**Figure 3:**
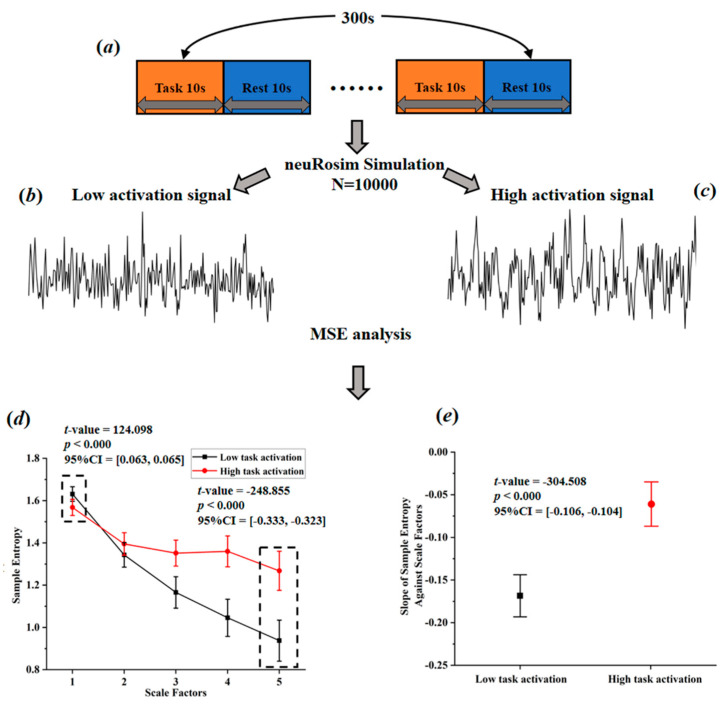
Simulated task-activated BOLD signals and multiscale entropy measures. (**a**) flow chart of stimulation task; (**b**) stimulated low activation signals; (**c**) simulated high activation signals; (**d**) entropy of the simulated signals over a range of scale factors; (**e**) the slopes of entropy measures against scale factors.

Based on a comprehensive consideration of physiological complexity and psychopathology, Yang et al. [49] proposed a revised theory of complexity, which can effectively explain the abnormally elevated complexity found in some studies. This theory suggests aging or neurophysiological disease may degrade mental function (Yang et al. [49] believe that individual mental function manifested in patterns of cognition, speech, behavior, and thought. For example, compared with normal people, the patterns of speech, behavior, and thought in AD patients show higher regularity, leading to a lower complexity of mental function in AD patients.) while reducing physiological complexity. The decrease of complexity can be manifested in two ways, one is toward regular pattern (reduced BOLD complexity due to the increased regularity), and the other is toward random pattern (reduced BOLD complexity due to the increased randomness). From this perspective, it is very likely that the abnormal high entropy values may actually reflect the decreased BOLD complexity toward random pattern. This theory, to some extent, can be tested through simulation. Sokunbi et al. [19] and Gu et al. [20] found that compared with healthy controls, patients with mental illness exhibited lower task-elicited brain activation but higher brain entropy. Based on these findings, we simulated brain activation for two groups (high activation signals simulated for healthy controls; low activation signals simulated for patients; the activation intensity of high activation group was set 10 times higher than that of low activation group) using neuRosim (NeuRosim is a R-based fMRI data simulator developed by Welvaert et al. [50] from Ghent University in Belgium. In addition, to simulate the brain activation under different tasks, it can also simulate system noises, temporal noises, low-frequency drifts, physiological noise, and task-related noise that exist in real task fMRI data.) and calculated the temporal multiscale entropy (MSE) value over a range of scales. As demonstrated in Figure 3d, when the scale factor was set to 1, the MSE (According to Yang et al. [26], the parameters of MSE were set as *m* = 1; *r* = 0.35; *l* = [1–5].) of the patient group was significantly higher than that of the control group, which was the same as reported in previous studies that the BOLD complexity was higher in patients. However, as the scale factor increased, the MSE of the control group gradually increased and became higher than that of the patient group. We also found a significant higher slope of entropy decay across time scales in the patient group than in the control group (Figure 3e). With the MSE measure, we could conclude that compared to high task-related activation group, the complexity of low task-related activation group showed a decreased complexity toward randomness. If previous research applied the MSE measure, it is likely that they may have found decreased complexity in brain activity of the patients. In a subsequent study, researchers found that different types of schizophrenia are associated with different patterns of BOLD complexity. Specifically, positive symptoms of schizophrenia were associated with a reduction in BOLD complexity toward regularity, while negative symptoms of schizophrenia were associated with a reduction in BOLD complexity toward randomness [26]. Then a third study adopting this method explored the altered complexity patterns among multiple groups; researchers found significant differences as well as overlaps of brain BOLD signal complexity between different psychotic disorder groups, suggesting the potential of categorizing psychosis based on such a complexity theory [11]. In sum, the theory proposed by Yang et al. [49] has well explained the abnormally high BOLD complexity and shown potentials for the future multiple group classification analysis. Yet, it has not been wildly applied in recent years due to the limitation of multiscale entropy algorithm, which will be introduced in Section 3.2.

In addition to the association between BOLD complexity and group traits, some researches explored the relation between BOLD complexity and other potential biomarkers. Song et al. [24] examined the relation between SampEn of BOLD signals and the fractional amplitude of low-frequency fluctuations (fALFF) in healthy adults and found no significant correlation in most brain regions, indicating that brain entropy and fALFF can provide independent information of brain activity. Wang et al. [31] investigated the relation between regional homogeneity (ReHo) and permutation entropy (PE) of BOLD signals in patients Alzheimer’s disease; they found no significant correlation in most areas except in disease-related regions, indicating that although brain entropy and ReHo are independent to each other, they are both able to provide effective information regarding the abnormality of the brain.

To sum up, brain BOLD signal complexity does have the potential to be an effective biomarker for different neurophysiological diseases. However, there also remain several challenges, which will be discussed in the next section.

### 3.2. Main Complexity Measures for Brain Blood-Oxygen Signals

**Figure 4:**
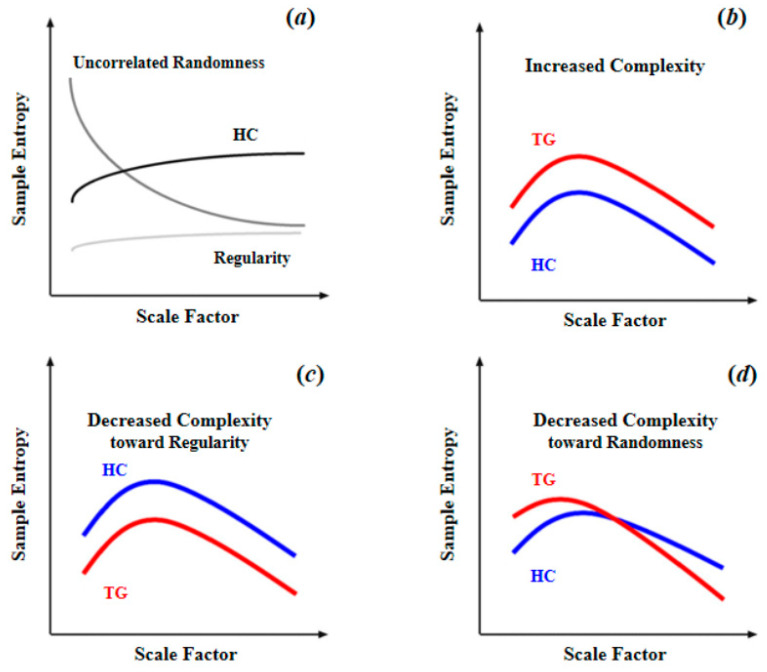
Schematic illustration of quantifying complexity of resting-state fMRI signal using multiscale entropy: (**a**) differences of MSE patterns across scale factors among the signals of uncorrelated randomness, HC, and regularity; (**b**) increased complexity as entropy increased in TG compared to HC across all scale factors; (**c**) decreased complexity toward regularity as reduced entropy in TG compared to HC across all scale factors; (**d**) decreased complexity toward randomness as entropy increased in fine time scale in TG compared to HC, and the entropy decayed as the scale factor increased. Reprinted with permission from ref. [11,26]. Copyright 2021 Elsevier.

As summarized in Table 1, for brain blood-oxygen signals, the most common complexity measures are approximate entropy (ApEn), sample entropy (SampEn), multiscale entropy (MSE), and permutation entropy (PE). ApEn originated from a theory that describes the regularity of a series of signals proposed by Pincus [51], which is closely linked to Kolmogorov entropy [52], a classical approach for determining the rate of information production. However, since ApEn lacks relative consistency and is heavily dependent on data length, Richman and Moorman [53] then developed sample entropy (SampEn) to reduce the biases of ApEn. Thus, SampEn can be considered as an improved algorithm based on ApEn. Multiscale sample entropy (MSE), another type of entropy described in this article, shares nearly the same core algorithm with SampEn. Compared with other entropy measures, MSE can provide information about complexity over a range of scales. For MSE, the scale factor determines the coarse-graining (coarse-graining means averaging a successive number of data points, as the scale factor increases, the number of consecutive data points increases, making the signals coarser on time scales.) level of signals. Over a range of scale factors, the profile of MSE has a consistent pattern with the relationship between regularity and randomness (as illustrated in Figure 4a, as scale factor increases, the BOLD MSEs of healthy controls gradually increase and eventually became higher than those of complete random or ordered signals), which can differentiate distinct signals. In addition, by comparing the profiles of MSE between target group (TG) and healthy controls (HC), changes in patterns of complexity (i.e., increased complexity; decreased complexity toward regularity or randomness (statistically, the criterion to determine the increased complexity (or decreased complexity toward regularity) of the target group is that, compared to healthy controls, the target group shows significant higher (or lower) MSE or mean MSE in all scale factors. The criterion to determine the decreased complexity toward randomness is that, compared to healthy controls, the target group shows significantly higher MSE in fine scale or significant lower MSE in coarse scale, and also shows a significant lower slope of entropy decay) of the target group could then be obtained. Compared to single-scale entropy measures, MSE can provide more information of brain complexity. However, from Table 1 we can find MSE was less often applied in previous studies. This is because in practical applications, an important limitation of MSE statistic is its requirements of sufficient sampling time points, which is undoubtedly challenging to fMRI data of low temporal resolution. For example, Yang et al. [26] performed an MSE analysis with a scale of 1–5 and 195 time points; such a length of time points was usually difficult to obtain for many fMRI studies due to the limitation of the temporal sampling rate of MRI. However, with an increasing application of fNIRS to obtain blood-oxygen signals of high temporal resolution, MSE may become a promising complexity analytical tool in future studies. As for permutation entropy (PE), in algorithm, it is close to classical Shannon entropy [31]. In practice, it is close to Lyapunov exponents and is particularly suitable for observational and dynamical noise [54]. Because of the essential differences between PE and three other types of entropy, direct comparison cannot be conducted. However, it is clear that as a type of single-scale entropy, the ability of PE to describe signal complexity is inferior to MSE. To sum up, the MSE with multiple scale factors is the best approximation of brain complexity, thus it should be preferred if the data is of enough sampling time points.

### 3.3. Optimizing Parameters for Complexity Measures

As summarized in Table 1, in practice, there are three common ways to optimize parameters for complexity measures. The first and most common approach is the empirical approach. Based on previous work and data length, the empirical value for parameters could be roughly selected and the recommended range of parameters listed in Table 2 are determined by this method. The second approach is ‘maximizing between-group difference’ approach, that is, to find a combination of parameters that maximize the differences of BOLD complexity between two distinct groups [16,24]. However, as one study pointed out, being able to show greater differences between groups does not guarantee that those parameters are less free from error or bias [25]. The third approach is to minimize the relative error of the entropy of BOLD signal in cerebrospinal fluids (CSFs), which contained minimal physiologic information but uncorrelated noise [32]. However, this approach can only be applied to whole brain BOLD data where signals from a large amount of cerebrospinal fluids are obtained. At present, the empirical approach has been used most frequently in studies because compared with the other two approaches, it was less complicated. From a scientific point of view, however, when analyzing whole brain BOLD signals, the approach of minimizing relative error in CSFs should be preferentially used to determine the optimal parameters for complexity measures.

## 4. Future Directions

### 4.1. Improving Brain Blood-Oxygen Signal Complexity Measurement

A set of precise and reliable analytical tools and methods are very important for a field of research. However, at present, studies aiming to improve methodology in complexity measurement are still lacking. Therefore, it is important to develop analytical techniques and research methodology for brain blood-oxygen signal complexity. Based on the above analysis, this paper presents the following suggestions.

First, it is critical to develop a novel analytical method that is suitable for brain complexity. Current complexity algorithms are mostly univariate based; when calculating brain complexity in a certain location, the influences from adjacent locations have not been duly considered. The current way does not take full advantage of the high spatial resolution of the BOLD signals. Besides, some analytical methods that consider BOLD signals in adjacent location have been successfully applied, such as regional homogeneity (ReHo) and multi-voxel pattern analysis (MVPA), which is an analytical approach based on spatial pattern formed by multiple voxels, and has recently been widely used because it can overcome the limitation of low signal-to-noise ratio and stringent multiple testing corrections brought by conventional voxel-wise analysis to some extent [57]. Therefore, a new complexity analytical method that consider multi-voxel blood-oxygen signal patterns is promising.

Second, the latent variable analysis can be used to describe complexity. On one hand, in previous studies, brain entropy values were often seen as direct representations of brain complexity. However, as mentioned in Section 2, the complexity of BOLD signals is more like a latent trait that cannot be directly measured through certain algorithms. Thus, it is more appropriate to take brain BOLD complexity as a latent variable and the entropy value calculated from various algorithms as corresponding manifest variables. On the other hand, to examine the intricate relation between the brain and individual differences, more flexible analytical methods are required, and the psychometric quality should be carefully assessed in the interpretation of brain-traits relationship. With latent variable analytics, researchers are allowed to incorporate known sources of between-subject variance (e.g., demographic characteristics, distinct metrics of latent traits, measured behavioral data, and brain complexity) into simple [58], theoretically-specified models, ultimately forming a comprehensive understanding of the relation between BOLD complexity and individual traits.

### 4.2. Accurate Trait Classification Methods

One of the core objectives of complexity studies is to distinguish patient groups with different traits based on the patterns of brain complexity. So far, only a limited number of studies have addressed the issue of prediction and classification. A recent study used SampEns of brain as features, to build a machine-learning classifier to differentiate patients with ASD from typical children [40]. This study represents a future development trend applying machine-learning techniques to classify complexity patterns of brain blood-oxygen signals from different patient populations. Moreover, because the human brain is a highly complex system, no single metric can provide comprehensive information of the brain. Also, previous studies have shown that brain entropy provides unique information that has little overlap with other potential biomarkers (e.g., ReHo, fALFF) [31,38]. Therefore, in future research, various metrics such as brain complexity, ReHo, and fALFF could be combined to generate a multidimensional feature and make the classification more reliable and precise.

### 4.3. The Dynamics of Blood Oxygen Signals Complexity

As mentioned above, the whole-brain distribution patterns of BOLD complexity in rest and task states are different. However, how the distribution patterns change dynamically between these two states remains unexplored. Future studies may build upon analytics of dynamical functional connectivity [59] to investigate the dynamics of BOLD complexity. In order to give a better description of the dynamic process, the blood-oxygen signal data should have sufficient time points. Therefore, the blood-oxygen signals of high temporal resolution, such as signals obtained by fNIRs, could be an ideal choice.

## 5. Conclusions

For various neurophysiological diseases, a reliable and subjective biomarker is of great importance. Blood-oxygen signal complexity has the potential to become an ideal biomarker. However, as there are some misuses of complexity analysis in previous studies, blood-oxygen signals complexity measurements still need a set of standardized guidelines for the optimal selection of complexity algorithms as well as corresponding parameters. With future improvements in the approaches to complexity calculations, we believe such an issue can be solved and a comprehensive understanding of the relation between brain blood-oxygen signal complexity and related neurophysiological traits can be gained.

## Figures and Tables

**Table 2:** Main entropy algorithms applied in the analysis of BOLD complexity.

Category	Ref	Parameters	Recommended Range
ApEn	[51]	*m*, pattern length; *r*, tolerance value	$10^{m}<N<20^{m}$ 0.1∗SD<r<0.6∗SD
SampEn	[53]	*m*, pattern length; *r*, tolerance value	0.1∗SD<r<0.6∗SD
MSE	[55]	*m*, pattern length; *r*, tolerance value; *l*, scale factor	$l*10^{m}<N<l*20^{m}$ 0.1∗SD<r<0.6∗SD
PE	[56]	*m*, pattern length	(m+1)!<N

Note: *m* is a positive integer; *N* specifies the data length and is a positive integer; *l* is a positive integer larger than 1; *SD* is the standard deviation of signals.

## Data Availability

The R code for task-activated BOLD signals simulation and the Matlab code for further complexity analysis are available upon request.
